# Joint Genome-Wide Profiling of miRNA and mRNA Expression in Alzheimer's Disease Cortex Reveals Altered miRNA Regulation

**DOI:** 10.1371/journal.pone.0008898

**Published:** 2010-02-01

**Authors:** Juan Nunez-Iglesias, Chun-Chi Liu, Todd E. Morgan, Caleb E. Finch, Xianghong Jasmine Zhou

**Affiliations:** 1 Molecular and Computational Biology, University of Southern California, Los Angeles, California, United States of America; 2 Davis School of Gerontology, University of Southern California, Los Angeles, California, United States of America; Lehigh University, United States of America

## Abstract

Although microRNAs are being extensively studied for their involvement in cancer and development, little is known about their roles in Alzheimer's disease (AD). In this study, we used microarrays for the first joint profiling and analysis of miRNAs and mRNAs expression in brain cortex from AD and age-matched control subjects. These data provided the unique opportunity to study the relationship between miRNA and mRNA expression in normal and AD brains. Using a non-parametric analysis, we showed that the levels of many miRNAs can be either positively or negatively correlated with those of their target mRNAs. Comparative analysis with independent cancer datasets showed that such miRNA-mRNA expression correlations are not static, but rather context-dependent. Subsequently, we identified a large set of miRNA-mRNA associations that are changed in AD versus control, highlighting AD-specific changes in the miRNA regulatory system. Our results demonstrate a robust relationship between the levels of miRNAs and those of their targets in the brain. This has implications in the study of the molecular pathology of AD, as well as miRNA biology in general.

## Introduction

Neurodegeneration and dementia in Alzheimer's disease (AD) are associated with neurotoxicity of the amyloid-beta peptide, which accumulates as amyloid fibrils in senile plaques characteristic of AD, and as oligomers that directly bind to neurons [1]–[3]. The production and clearance of the amyloid-beta peptide is therefore a major target of investigation on the pathogenesis of AD and therapeutic interventions for prevention and treatment. With the exception of the rare familial forms caused by dominant mutations, the initiating factors in most cases of AD remain unresolved. A major clue was given by Down's syndrome, in which trisomy of chromosome 21 results in early onset of AD, but not in a case in which chromosome 21 had a mosaic deletion of the amyloid precursor protein (APP) locus [4]. This evidence highlighting the importance of gene dosage in AD is one of the rationales for the study of gene expression in AD brain.

Many studies have therefore examined changes in mRNA prevalence in the brain in later stages of AD (reviewed in [5]). There is, however, surprisingly little agreement between the differentially expressed gene lists of different studies. Furthermore, among the few RNA changes that are shared among expression studies, there is no clear functional relationship. A future challenge is to integrate large amounts of data in a network context, for which studies such as OSCAR [6] may be a useful model.

Besides mRNA populations, microRNAs (miRNAs) may also be important in AD. MicroRNAs are short non-coding RNAs (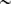
22nt long) that bind complementary sequences in target mRNAs and can thus cause their selective degradation, or selective inhibition of translation [7], [8]. Although miRNAs have been intensely studied in the context of cancer progression, their role in AD has received less attention [9]–[12]. The most detailed of the existing studies [10] showed decreased levels of miR-29a/b in AD, which was predicted to cause increased levels of beta-amyloid cleaving enzyme 1 (BACE1), an essential protein in the generation of beta-amyloid from APP, and this prediction was confirmed in vitro. Using oligonucleotide arrays followed by targeted experiments, Wang *et al* [12] showed decreased levels in AD of miR-107, which also targets BACE1.

In the present study, we used microarrays to simultaneously measure the levels of miRNA and mRNA in the parietal lobe (Pl) cortex of AD patients and age-matched controls. Prior studies of specific miRNAs suggested that some miRNAs directly decrease the levels of target mRNAs on a genome-wide scale [13]–[16]. Starting from genome-wide miRNA and mRNA expression data, we devised a novel permutation scheme to robustly determine the significance of any correlation between levels of miRNAs and their target mRNAs. Although the levels of around 20 miRNAs showed a significant negative correlation with those of their targets, we were surprised to find a much larger number, over 50, *positively* correlated with their targets. We further showed that mRNAs involved in specific processes, such as fatty acid metabolism and protein refolding, are responsible for this correlation signal, and confirmed that these processes are specific to the brain by comparison to a publicly available dataset of cancer cell lines [17] and one of primary breast cancer cells [18]. Finally, using the permutation approach *separately* in the AD and in the control samples, we were able to detect a large set of changes in the miRNA-target correlations in AD brain. This points to changes in the miRNA regulatory system in Alzheimer's disease.

## Results

### Differentially Expressed miRNAs and mRNAs

Total RNA was extracted from the parietal lobe cortex of 10 individuals (5 AD patients and 5 age-matched control subjects). After RNA quality control, however, only 8 samples (4 from each group) were used in the final study. Although these are too few to draw any reliable conclusions about differential expression of hundreds of miRNAs and mRNAs, we briefly present that analysis for completeness and as a resource for future meta-analyses. We used the empirical Bayes procedure [19] to determine the statistical strength of possible changes in RNA prevalence, and found 48 differentially expressed miRNAs and 11 differentially expressed mRNAs at a False Discovery Rate [20] of 0.05 (see Table S1 and Table S2).

For the functional analysis of the differentially expressed miRNAs, we predicted the functions of miRNAs to be the functions overrepresented among their targets (hypergeometric p-value 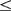
0.01). The predicted functions can be found in Table S3 (biological process), Table S4 (molecular function), and Table S5 (cellular component). Overrepresented functions among significantly upregulated miRNAs included plasma membrane (p-value: 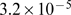), cell adhesion (p-value: 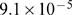), transmembrane receptors (0.0065), and transmembrane transporters (0.0086).

No overrepresented functions were found among the differentially expressed mRNAs.

### Correlation between Expression Levels of miRNAs and Target mRNAs

Much evidence shows that mammalian miRNAs, like their plant counterparts, can influence gene expression not only by inhibiting protein translation, but also by causing the degradation of their target mRNA [14]–[16]. We therefore looked for negative correlation between the expression levels of miRNAs and their predicted target mRNAs. For individual miRNA-target pairs, our data did not support this hypothesis: after multiple-testing correction, no pairs passed the 0.05 FDR level.

**Figure 1:**
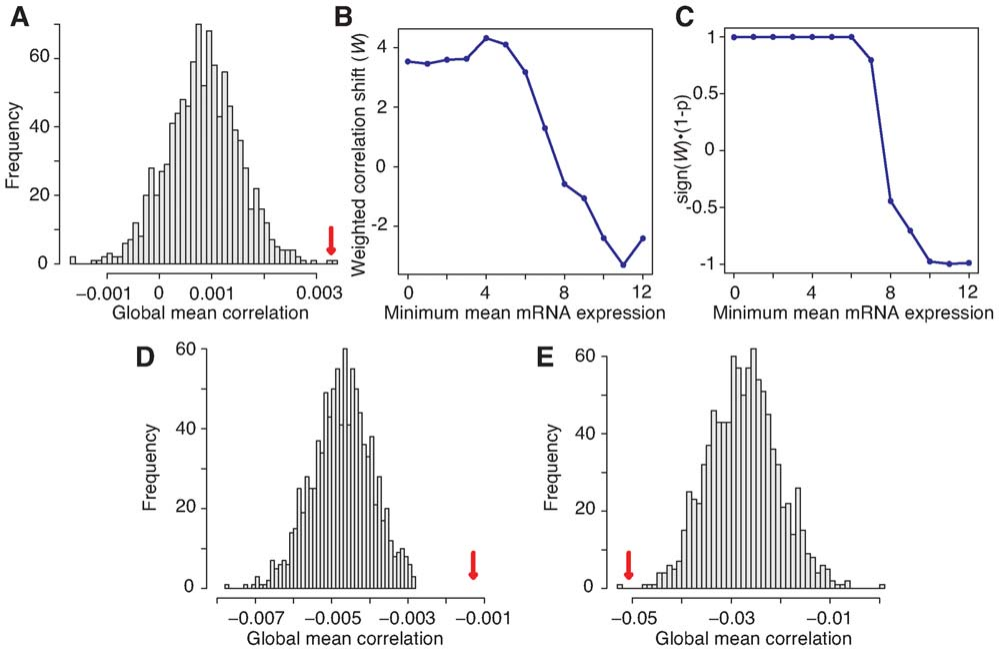
Permutation of miRNA-mRNA target relationships reveals a positive correlation between miRNAs and their targets, or negative for some high-prevalence mRNAs. (A) Histogram of permuted values of mean correlation between levels of miRNAs and those of their target mRNAs. Red arrowhead indicates true value. (2-sided p-value: 0.002) (B), (C) Weighted correlation shift (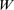) and 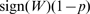 for a range of cutoffs of log (base 2) mRNA mean expression. (D), (E) Histogram of permuted values for mRNA log-expression cutoffs 4 and 11. (All plots were generated using a ddG cutoff of 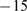 kcal/mol.)

We then tried to detect potentially subtle correlations by deriving global statistics about miRNA-target expression correlations. If high levels of a miRNA were degrading some target mRNAs, we would expect that, among all miRNA-target expression correlations, there would be a measurable bias towards negative correlations. To test this, we calculated the mean expression correlation among all miRNA-target pairs, then repeated the calculation after randomizing the miRNA targeting predictions. Instead of a negative correlation bias, this permutation analysis showed a significant bias towards positive correlations (Figure 1A). The average correlation among 240,758 miRNA-target pairs in our data (with a ddG threshold of 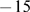 kcal/mol for PITA) was 0.00328, compared to a mean correlation among permuted pairs of 0.000836. This shift had a two-sided empirical p-value of 0.002 among 1,000 permutations. (These results are qualitatively identical over a range of ddG thresholds; see Figure S2.)

We were initially surprised to find positive correlations between levels of miRNAs and their target mRNAs. Because two recent studies [13], [21] found that, for some miRNAs, miRNA levels affect target mRNA levels only for high-prevalence mRNAs, we repeated the analysis, but using only mRNAs with relatively high average expression values. Indeed we found a striking change from a positive correlation shift over all miRNA-target pairs to a negative correlation shift for miRNA-target pairs restricted to mean mRNA expression levels of 11 or greater (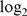 scale) (p-value  =  0.002 after 1000 permutations) (Figure 1) (log mRNA expression levels range from 2 to 15; see Figure S1 for the full distribution).

### Individual RNAs Involved in the Correlation Shift

We detected an overall bias in miRNA-target correlations towards positive correlations, relative to a permuted distribution. We wished to determine whether the bias was due to a few miRNAs having a strong correlation with their targets, or most miRNAs having a weak correlation with their targets. In the first case, we would expect an essentially uniform distribution of p-values with a strong peak near zero. In the second case, we would expect a skewed p-value distribution.

**Figure 2:**
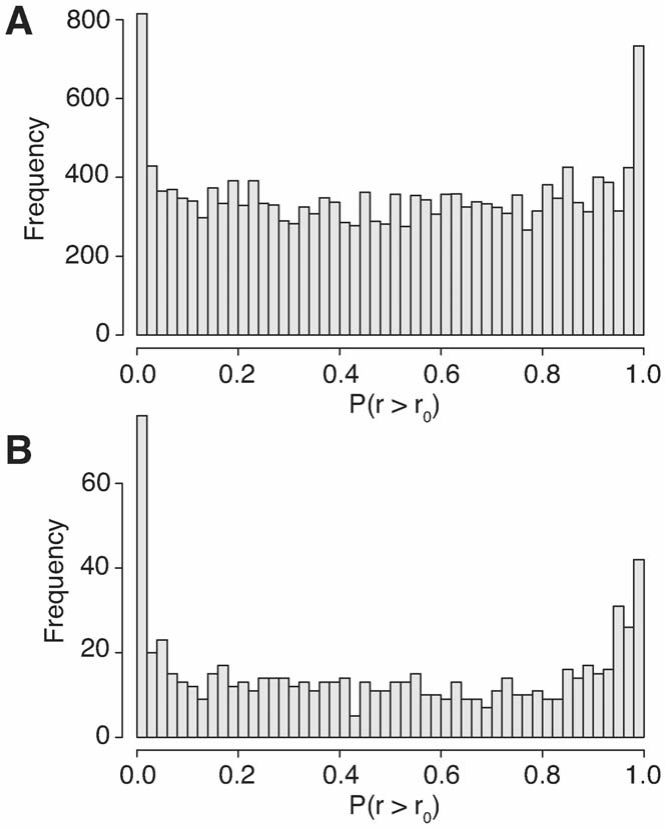
Histograms of miRNA- and mRNA-level p-values. mRNA (RefSeq) (A) and miRNA (B) p-value histograms using mRNA log-expression cutoff 4. The p-values are from 1,000 permutations. Here, 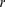 is the observed mean correlation for that RNA, and 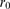 is the corresponding permuted correlation.

The observed distributions of empirical p-values for both miRNAs and mRNAs are shown in Figure 2. (An equivalent figure using the TargetScan miRNA target prediction method is shown in Figure S3, which shows that this result is robust with respect to the prediction method used.) These histograms reveal that most miRNA-mRNA pairs are actually uncorrelated, with only about 60 miRNAs and 400 mRNAs contributing to the positive correlation bias detected among all pairs. Interestingly, despite a positive correlation *on average* among all miRNA-target pairs, we also found strong peak of about 20 *negatively* correlated miRNAs and 300 negatively correlated mRNAs. Therefore, the positive correlation average masks distinct populations of positively and negatively correlated miRNA-target pairs, and it is the relative abundance of positive and negative correlations that drives the average.

Having determined that only relatively few miRNAs actually exhibit correlations with their targets (whether positive or negative), we show the 10 most positive miRNA-target pairs in Table 1 and the 10 most negative pairs in Table 2. Note that no correspondence should be inferred between the miRNAs and mRNAs in these two tables: the presence of an RNA in either table is the result of summing over many correlations. Since miRNAs can target hundreds or even thousands of mRNAs, no individual correlations can by itself much affect the 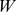-score, and conversely, a high 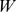-score does not imply high correlation across *all* a miRNA's targets. The full data of weighted correlation shifts can be found in Tables S6 and S7. Additionally, we used miRNA-mRNA concordance (see Methods, “MicroRNA-mRNA mutual concordance score and differential mutual concordance score” below) to generate pairs of miRNA-targets in which both RNAs had high 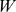-scores *and* were highly correlated to each other (Table S8).

**Table 1:** Most positively correlated mRNAs and miRNAs.

mRNAs							
**RefSeq ID**	**Gene ID**	**Gene Symbol**	**mean**	**n**	**rand**	**p**	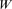
NM_015723	50640	PNPLA8	0.174	33	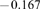	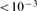	6.35
NM_152487	148534	TMEM56	0.337	14	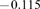	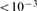	5.88
NM_007173	11098	PRSS23	0.138	28	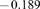	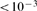	5.85
NM_004849	9474	ATG5	0.088	51	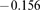	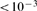	5.71
NM_006500	4162	MCAM	0.349	80	0.103	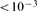	5.60
NM_006597	3312	HSPA8	0.841	4	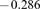	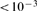	5.55
NM_015235	23283	CSTF2T	0.034	50	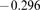	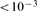	5.50
NM_001017963	3320	HSP90AA1	0.439	12	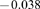	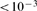	5.22
NM_001199	649	BMP1	0.289	50	0.053	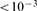	5.01
NM_018710	55529	TMEM55A	0.408	8	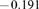	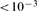	5.01

The ten mRNAs most positively correlated with their regulating miRNAs, and ten miRNAs most positively correlated with their target mRNAs, as measured by their weighted correlation shift (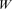). Repeated mRNAs (RefSeq IDs that map to the same UTR and therefore the same probesets and same expression) were removed from the table. Data was limited to mRNAs with log-expression values greater than 4. **Header legend:** *mean*: mean correlation over all targets (in the case of miRNAs) or regulators (in the case of mRNAs); *n*: number of correlations included in the mean. *rand*: average correlation after randomization.

**Table 2:** Most negatively correlated mRNAs and miRNAs.

mRNAs							
**RefSeq ID**	**Gene ID**	**Gene Symbol**	**mean**	**n**	**rand**	**p**	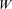
NM_014810	9857	CEP350	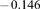	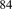	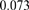	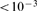	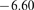
NM_003012	6422	SFRP1	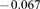	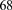	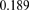	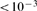	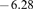
NM_052854	90993	CREB3L1	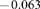	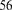	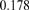	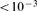	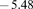
NM_006499	3964	LGALS8	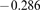	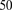	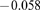	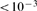	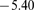
NM_201543			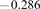	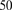	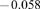	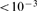	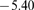
NM_000517	3040	HBA2	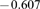	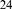	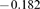	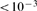	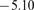
NM_000558	3039	HBA1	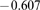	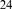	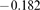	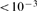	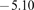
NM_004305	274	BIN1	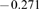	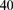	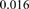	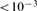	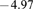
NM_000873	3384	ICAM2	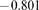	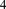	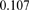	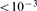	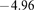
NM_001008540	7852	CXCR4	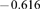	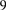	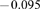	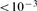	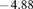

The ten mRNAs most negatively correlated with their regulating miRNAs, and ten miRNAs most negatively correlated with their target mRNAs, as measured by their weighted correlation shift (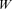). Repeated mRNAs (RefSeq IDs that map to the same UTR and therefore the same probesets and same expression) were removed from the table. Data was limited to mRNAs with log-expression values greater than 4. **Header legend:** *mean*: mean correlation over all targets (in the case of miRNAs) or regulators (in the case of mRNAs); *n*: number of correlations included in the mean. *rand*: average correlation after randomization.

To analyze the functions of those highly correlated (positively or negatively) miRNAs and mRNAs, we generalized the idea of weighted correlation shift from RNAs to groups of RNAs, in this case defined by GO biological processes. We then asked whether RNAs involved in some biological processes exhibit particularly high or low correlations. Among the processes most positively correlated with their regulating miRNAs, we found some very specific processes, including metabolism of both carbohydrates and fatty acids, as well as protein refolding, indicating the particular importance of these processes in the brain. Among the processes most negatively correlated are general regulatory functions such as RNA splicing and translational elongation (which could indicate an interesting self-regulatory loop in miRNA function), but also more specific processes that could play a role in AD brain, such as oxygen transport, cell adhesion, inflammatory response, cytoskeletal organization and dendrite development (see Table S9).

### Comparison to Other miRNA/mRNA Datasets

To further validate these new methods, we examined the correlation shift in two other miRNA/mRNA expression datasets: the NCI-60 cell line panel [17], and a panel of primary breast tumor cells [18].

**Figure 3:**
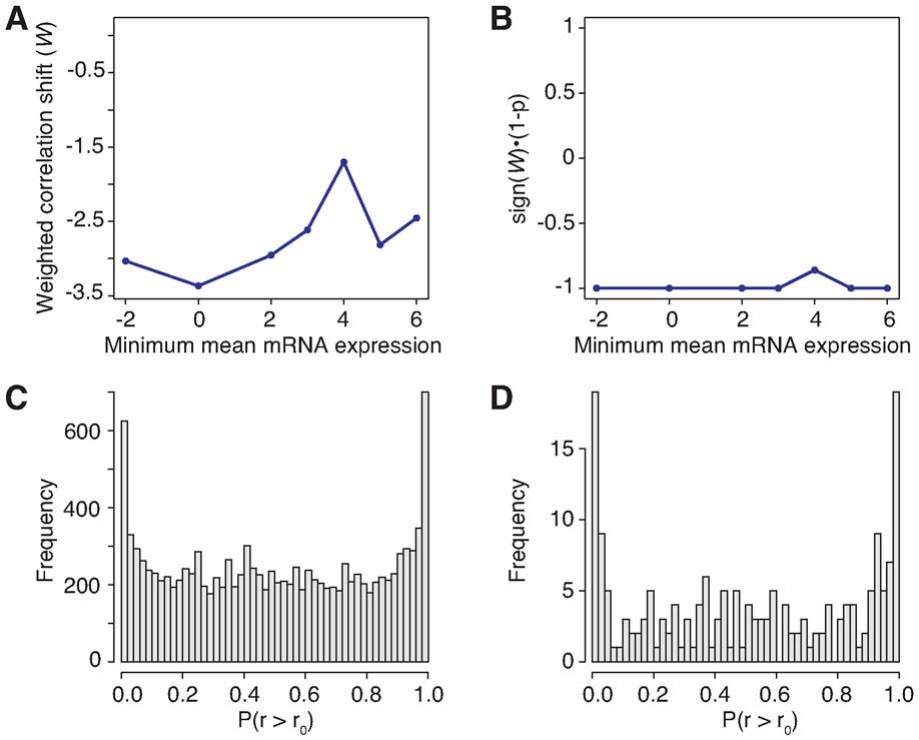
NCI-60 overall weighted correlation shift, significance, and p-value distributions. (A) Weighted correlation shift (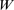) vs minimum log (base 2) mRNA expression value. (B) Significance (sign(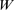)

The NCI-60 dataset displayed an overall negative correlation bias throughout the range of expression values (Figure 3; see Figure S4 for the distribution of expression values), and an entirely different set of miRNAs and Gene Ontology biological processes associated with the correlation bias. For example, the most highly correlated processes in the NCI-60 panel are “hepatocyte growth factor receptor signaling pathway”, “myoblast proliferation”, “regulation of cytokine-mediated signaling pathway”, “immune response-regulating signaling pathway”, “chemokine receptor transport within lipid bilayer”, “regulation of transcription from RNA polymerase II promoter by carbon catabolites”, and “UV protection” (Table S10). None of these are positively or negatively correlated in the brain samples. Note that even in this case, there are distinct miRNA-target populations that are either positively or negatively correlated (Figure 3, panels C and D); the overall average correlation is negative because the relative abundance of positively and negatively correlated pairs is inverted compared to that in our dataset. This result implies that our method can find active miRNA-mRNA relationships in their specific context, and that, as expected, these active relationships are different between cancer cell lines and brain cells.

(1-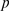)) vs minimum log (base 2) mRNA expression value for the NCI-60 cancer cell line dataset. (C), (D) Distribution of p-values of mRNA and miRNA

**Figure 4:**
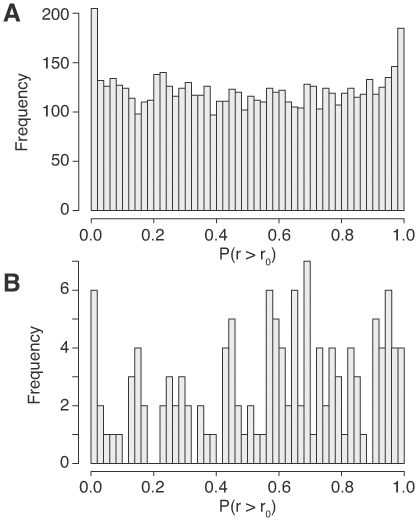
Primary breast tumor p-value distributions for mRNA (A) and miRNA (B) correlations.

In the primary breast tumor dataset, the average overall correlation was not significantly shifted in either the positive or negative direction (not shown). However, when we examined the distribution of p-values for mRNA weighted correlation shifts, we again found that a number of mRNAs were significantly positively correlated with their regulating miRNAs, while a similar number were significantly negatively correlated (Figure 4A). The histogram of p-values for miRNAs (Figure 4B) is too noisy to draw any conclusions from it, but this could be partly due to the low number of represented miRNAs in this dataset (130 vs 889 in our brain dataset and 189 in the NCI-60 dataset).

Taken together, these results suggest that miRNA-target correlations would be detectable in most expression datasets using our method, as we have found evidence of these correlations in three completely independent datasets. The main difference between datasets then seems to be whether positive or negative correlations predominate, or neither.

### Regulatory Changes between miRNAs and Target mRNAs in Alzheimer's Disease

The correlation shifts discussed so far are exhibited across all AD and control samples. To identify specific correlations that may have been gained or lost in AD brain, we repeated the permutation analysis separately on the AD samples and the control samples, to look for the largest changes in weighted correlation shifts. Note that this analysis is independent of changes in expression between the two sample groups; it instead detects changes *between* the groups in correlations found *within* the groups, and can thus indicate changes in miRNA function in the AD brain. To compensate for small sample size, we exploited the many-to-many network of miRNA targeting relationships to create a differential mutual concordance score (see details in Methods) and thus identify the miRNA-target mRNA pairs exhibiting the highest correlation change between AD and control. We specifically searched for pairs that were concordant (positively or negatively correlated with each other and with other targets/regulators) in AD but not in control samples, and vice versa. These pairs should indicate regulatory relationships being turned on and off in AD brain.

A Mann-Whitney test comparing the ranks of pairs containing AD-related genes (defined by GeneRIF [22]) to those containing genes unrelated to AD gave a p-value of 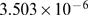, indicating that differential concordance analysis can recover miRNA-target relationships that are functionally relevant to AD. The 40 most changed regulatory pairs are represented as a network in Figure S5 (the full results can be found in Table S11).

## Discussion

This is the first study of AD to measure mRNA and miRNA levels in the same brain samples and supports the potential role of miRNAs in AD pathogenesis. We found a substantial number of new differentially expressed miRNAs at an FDR of 0.05. The expression of 5.4% of sampled miRNAs was affected by AD at this statistical significance level, suggesting a new approach to the molecular analysis of AD. We found a strong concordance between the miRNAs with differential expression in AD cortex as found in our study and in that of Hébert *et al* [10]. Of the 16 miRNAs they reported, 5 were found in our set of 29 differentially expressed (non-predicted) miRNAs, and all in the same direction (p-value: 0.0084). Results on differentially expressed miRNAs in AD [9], [23], [24] appear to be more robust and reproducible than those of differentially expressed mRNAs [5].

Our joint miRNA-mRNA data and permutation analyses add to the mounting evidence that miRNAs can regulate the expression levels of target RNAs in humans. Recently, Vasudevan *et al* [25] found that miRNAs can switch from repression to activation of target translation, depending on the cell cycle state of the cell. Based on our analysis of data in the brain (containing predominantly cell cycle arrested cells) compared to the NCI-60 cell line data and primary breast tumor data (which consist of actively replicating cells), we propose that this switch could occur not only at the level of translation, but also at the level of mRNA stability. Further, individual miRNAs appear to have different mode of actions, since all three datasets present both positively and negatively correlated miRNAs. Our data, however, cannot distinguish between the regulation of mRNA levels by miRNAs and the coregulation of both miRNAs and their targets by upstream factors, as hypothesized by Tsang *et al* [26], who also found both positive and negative correlations in their data. Distinguishing between these two possibilities presents an exciting avenue for future research.

In any case, it is clear that there are at least two distinct populations of miRNA: one that positively correlates with its targets, and another that negatively correlates with its targets. Recent studies corroborate this finding [27], [28]. The targets of the latter population appear to include high-prevalence mRNAs that function in general transcription and translation machinery. Few other studies have jointly analyzed miRNA and mRNA array data, especially in the field of AD. Notably, miR-34c, for which we have strong evidence of positive correlation with its targets (Table 1), was also found to be positively correlated with its targets in mouse brain [26] and in rat oligodendrocytes [29]. miR-218, also reported in the above two studies, was not among our most highly positively correlated miRNAs, but did have a high positive weighted correlation shift (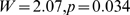). These may represent cases of miRNA and mRNA coregulation in the brain that are conserved in mammalian species, and should therefore be considered high priority targets for future studies.

Finally, our findings of miRNA-mRNA pairs differentially correlated between normal and AD brains point to a fine grained level of regulation of miRNA function. Just as transcription factor binding to target promoters has turned out to be condition-specific, the same could be true of miRNA-target UTR pairs. Our results demonstrate that aggregating correlation values from many different miRNA-target pairs, and calibrating these by permutation, is an effective scheme to computationally detect relationships between the levels of miRNAs and those of their targets on a genome-wide scale.

## Materials and Methods

### Tissue Samples and RNA Extraction

#### Ethics statement

Postmortem human brain samples were obtained from the USC Alzheimer's Disease Research Center (ADRC), which assures written informed consent from all subjects. The USC Institutional Review Board approved the use of the samples for this study.

#### RNA extraction

RNA was extracted using TriReagent from parietal lobes of postmortem brains of five subjects with Alzheimer's disease and five matched controls. The average age of subjects with Alzheimer's disease was 85 years (range 75 to 92 years) and 91.8 years (90 to 95 years) for controls. The postmortem interval ranged from 3.75 to 10.1h with a mean of 5.87h. Per-sample details can be found in Table S12, and in the Gene Expression Omnibus (see “Data Availability” below). We treated RNA samples with DNase and purified them with RNeasy mini columns (QIAGEN).

### mRNA Measurements

The mRNA array measurements were performed at the UCLA microarray core (http://microarray.genetics.ucla.edu) and used Affymetrix HG-U133 Plus 2.0 arrays.

### miRNA Measurements

MicroRNAs were assayed by LC Sciences (Houston, TX, USA, http://www.lcsciences.com) using a custom 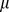-Paraflo array containing probes for 470 miRNAs from Sanger miRBase [30]–[32] and 419 miRNAs predicted by miRNAMap [33]. (Our entire analysis pipeline was repeated without the predicted miRNAs, and the results are qualitatively identical — see supplementary materials.)

As quality control, we verified our miRNA measurements with smiRNAdb [34], a mammalian miRNA expression atlas generated by library sequencing. There is a striking agreement between our parietal lobe measurements and their frontal lobe measurements (an exact tissue match was not available). Of the top 25 most highly expressed miRNAs in our data, 15 were in the top 25 in the atlas dataset, and 23 were in the top 50. The strong agreement between our expression values and those generated by an entirely different quantitation method gave us confidence in the quality of our data. In addition, we manually investigated several “brain-specific” and “brain-expressed” miRNAs reported in the literature, and consistently found them to show high expression levels in our data. For example, miR-124a had a mean expression level of 14.4 (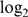 scale), while the full range of expression values is 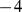 to 16 (see Figure S1). Other examples include miR-9 with a mean expression level of 15.7, miR-146a with 9.62, miR-134 with 9.81, and miR-195 with 13.2.

### Normalization and Analysis

We used the affy package in Bioconductor [35] to read in and normalize the microarray measurements for each RNA type. We used the robust multi-array average (RMA) normalization method [36], which consists of three steps: background correction, quantile normalization (each performed at the individual probe level), and robust linear model fit using log-transformed intensities (at the probeset level). We statistically evaluated changes in RNA prevalence by the empirical Bayes (eBayes) method [19] from the limma Bioconductor package. In a survey of methods that estimate differential expression, Smyth [37] showed that empirical Bayes improves specificity while having very little effect on sensitivity.

### miRNA Target Prediction

We predicted targets of both miRBase and miRNAMap miRNAs using the Probability of Interaction by Target Accessibility (PITA) method [38] developed by the Eran Segal lab. We used a ddG cutoff of 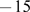 for most of the presented work, but also verified our results using a wider range of cutoffs (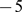 to 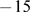). If there were multiple sites in a mRNA UTR, we selected only the one with the lowest free energy change. Following the original publication, we used a 3 nucleotide upstream flank and a 15 nucleotide downstream flank.

Repeating our study with predictions by TargetScan (context score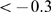), PicTar (score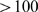) or Miranda (score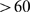) did not affect the general results of this paper, with the exception of the shift to an overall negative average correlation for high-prevalence mRNAs.

### MicroRNA Target Permutation Analysis

Our strategy for permutation first discretized the miRNA-mRNA target relationships by applying a ddG cutoff to the PITA predictions. In most presented analyses the cutoff was 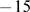 kcal/mol, but we showed that our results hold for a range of thresholds (see Figure S2). We converted miRNA-RefSeq pairs (as returned by PITA) to miRNA-probeset_id by using the HGU133 Plus 2.0 NA-27 table provided by Affymetrix.

The data can then be considered a bipartite graph, with nodes representing miRNAs on one side and probesets on the other, and edges representing PITA target prediction relationships. Nodes have associated expression measurements. We then computed statistics both globally (the mean correlation over all edges) and for specific subsets of nodes (for example, the mean correlation over all edges incident on one miRNA, or all edges incident on any probe set annotated with a particular GO function). Finally, we permuted the network by shuffling the edges, maintaining source and target node degrees, but without disallowing double edges, and recomputed statistics after shuffling. From these we can obtain an empirical p-value and a “weighted correlation shift”, 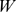, which we define as the difference between the true value and the mean permuted value, divided by the standard deviation of the permuted values:
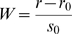
The weighted shift is identical to a 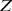-score, but using permuted rather than known/parametric mean and standard deviation.

### MicroRNA-mRNA Mutual Concordance Score and Differential Mutual Concordance Score

To focus on the most specific regulatory changes in AD, we searched for miRNA-target mRNA pairs exhibiting a high scoring correlation shift by combining the weighted correlation shifts (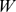) of miRNAs with those of their targets. We define the mutual concordance score of a miRNA-mRNA pair as the sum of their weighted correlation shifts, divided by 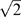. (This is the expected standard deviation of the sum of two independent Gaussian random variables.) We then identified miRNA-target mRNA pairs showing the highest difference in mutual concordance score between AD and control (which, again normalized by 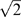, we called differential mutual concordance score). To maximize specificity, we only reported pairs with high differential mutual concordance scores that met two additional conditions. First, the miRNA and mRNA should both individually have high 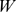-scores in either AD or control samples, to prevent a single RNA with an exceptionally high 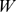 from appearing concordant with *any* target/regulator. And second, the correlation between an miRNA and target mRNA should be in the top 30% of correlations involving each of the RNAs, since, in an uncorrelated miRNA-target mRNA pair, the miRNA could be correlated with other targets, and the mRNA could be correlated with other regulating miRNAs, resulting in a high mutual concordance score when in fact the pair is discordant.

Note that this approach is more robust than merely calculating the correlations between miRNAs and their targets, because those are sensitive to biases in the samples, especially when the number of samples is limited. In contrast, our 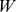-score is calibrated by permutation, eliminating many potential sources of bias in the expression data. Our mutual concordance score is also more powerful, since it pools information from many miRNA-target pairs to find highly correlated individual pairs. To illustrate this, a previous study of correlations in the NCI-60 dataset was unable to find any correlations reaching significance [17], while our permutation method did (see “Analysis of a cancer cell line dataset”, in Results).

The differential concordance network (Figure S5) was visualized using Cytoscape [39].

### Data Availability

The expression data generated by this study are available in the NCBI Gene Expression Omnibus (GEO) as accession GSE16759.

## Supporting Information

Figure S1 Histograms of log (base 2) expression values for mRNA and miRNA microarray data.
(0.42 MB EPS)

Figure S2 Significance of the correlation shift (sign(W)⋅(1-p)) as a function of the ddG cutoff and the mean mRNA expression cutoff (log-base-2 scale).
(0.52 MB EPS)

Figure S3 Histograms of miRNA- and mRNA-level p-values using TargetScan as the miRNA target prediction method. mRNA (RefSeq) (A) and miRNA (B) p-value histograms using mRNA log-expression cutoff of 4. The p-values are from 1,000 permutations. Here, r is the observed mean correlation for that RNA, and r_0 is the corresponding permuted correlation.
(0.46 MB EPS)

Figure S4 Histograms of log (base 2) expression values for mRNA and miRNA microarray data in the NCI-60 dataset.
(0.44 MB EPS)

Figure S5 MicroRNA-mRNA regulatory changes changed between normal and AD brain. The edge color represents the normalized difference between the mutual concordance scores in control and AD samples. Red means the concordance was more positive in AD than control, and green indicates it was more negative in AD. The edge shape is determined by the state in AD: an arrowhead indicates a positively correlated concordant pair in AD, a dashed line indicates a non-concordant (uncorrelated) pair in AD, and a flat arrowhead indicates a negatively correlated concordant pair in AD.
(0.55 MB EPS)

Table S1 MicroRNAs differentially expressed at the 0.05 FDR level.
(0.00 MB TXT)

Table S2 Messenger RNAs differentially expressed at the 0.05 FDR threshold. (Tab-delimited table.)
(0.00 MB TXT)

Table S3 Predicted miRNA GO Biological Processes. (Tab-delimited table. Col1: miRNA, Col2: GO Term ID, Col3: GO Term name. The ddG cutoff for PITA miRNA target prediction was −15kcal/mol, and the p-value cutoff for functional enrichment with the hypergeometric test was 0.01.)
(0.03 MB TXT)

Table S4 Predicted miRNA GO Molecular Functions. (Tab-delimited table. Col1: miRNA, Col2: GO Term ID, Col3: GO Term name. The ddG cutoff for PITA miRNA target prediction was −15kcal/mol, and the p-value cutoff for functional enrichment with the hypergeometric test was 0.01.)
(0.13 MB TXT)

Table S5 Predicted miRNA GO Cellular Components. (Tab-delimited table. Col1: miRNA, Col2: GO Term ID, Col3: GO Term name. The ddG cutoff for PITA miRNA target prediction was −15kcal/mol, and the p-value cutoff for functional enrichment with the hypergeometric test was 0.01.)
(0.08 MB TXT)

Table S6 RefSeq-level permutation results. (Tab-delimited table. Permutation used only mRNAs with mean log-expression greater than 4.)
(1.51 MB TXT)

Table S7 MicroRNA-level premutation results. (Tab-delimited table. Permutation used only mRNAs with mean log-expression greater than 4.)
(0.07 MB TXT)

Table S8 MicroRNA-mRNA highly concordant pairs.
(7.10 MB TXT)

Table S9 Permutation results for Gene Ontology biological processes. (Tab-delimited table. Permutation used only mRNAs with mean log-expression greater than 4.)
(0.61 MB TXT)

Table S10 Permutation results for Gene Ontology biological processes in the NCI-60 dataset. (Tab-delimited table. Permutation used only mRNAs with mean log-expression greater than 0.)
(0.90 MB TXT)

Table S11 Differential concordance analysis table, including edge ID and differential concordance scores. (Tab-delimited table.)
(5.49 MB TXT)

Table S12 Information about the biological samples used in the study.
(0.00 MB TXT)
